# Palbociclib exposure in relation to efficacy and toxicity in patients with advanced breast cancer

**DOI:** 10.1016/j.esmoop.2025.104290

**Published:** 2025-02-15

**Authors:** S.M. Buijs, M.I. Mohmaed Ali, E. Oomen-de Hoop, C.L. Braal, N. Wortelboer, A. van Ommen-Nijhof, G.S. Sonke, I.R. Konings, A. Jager, N. Steeghs, H. Siebinga, R.H.J. Mathijssen, A.D.R. Huitema, S.L.W. Koolen

**Affiliations:** 1 Department of Medical Oncology, Erasmus MC Cancer Institute, Rotterdam, The Netherlands; 2 Department of Pharmacy & Pharmacology, The Netherlands Cancer Institute, Amsterdam, The Netherlands; 3 Department of Medical Oncology, The Netherlands Cancer Institute, Amsterdam, The Netherlands; 4 Department of Medical Oncology, Amsterdam University Medical Center, Cancer Center Amsterdam, Amsterdam, The Netherlands; 5 Department of Medical Oncology, University Medical Center Utrecht, Utrecht, The Netherlands; 6 Department of Clinical Pharmacy, University Medical Center Utrecht, Utrecht University, Utrecht, The Netherlands; 7 Department of Pharmacology, Princess Máxima Center for Pediatric Oncology, Utrecht, The Netherlands; 8 Department of Pharmacy, Erasmus University Medical Center, Rotterdam, The Netherlands

**Keywords:** breast cancer, CDK4/6 inhibitors, pharmacokinetics, adverse events, dose reduction

## Abstract

**Background:**

Data on exposure–response or exposure–toxicity relationships of cyclin-dependent kinase 4/6 inhibitors (CDK4/6i) are limited and inconclusive. We aimed to investigate whether there is an association between palbociclib exposure and progression-free survival (PFS), adverse events (AEs) and dose reductions.

**Materials and methods:**

Data were retrieved from the prospective, multicentre SONIA trial in which patients with advanced estrogen receptor-positive, human epidermal growth factor receptor 2-negative breast cancer were randomised to receive CDK4/6i treatment in first versus second line. Blood for pharmacokinetics (PK) was taken at day 15 of cycles 1 and 2 during CDK4/6i treatment. Individual trough concentrations and plasma area under the curves of palbociclib were constructed using a population PK model. Associations with palbociclib exposure were tested using Cox regression for PFS and chi-square tests for AEs or dose reductions.

**Results:**

PK data were available for 344 patients. No association between palbociclib exposure and PFS was found. Although patients with higher palbociclib exposure had more dose reductions during their entire CDK4/6i treatment course, this was not reflected by a higher incidence of grade 3-4 AEs in the first 3 months.

**Conclusion:**

The absence of an association between palbociclib exposure and PFS and the presence of the association between palbociclib exposure and dose reductions suggest that dose reductions may safely be carried out in case of palbociclib-related toxicity.

## Highlights

• Up to now, data on exposure–response or exposure–toxicity relationships of CDK4/6i are limited and inconclusive.
• Patients with higher palbociclib exposure more frequently underwent dose reductions.
• No relationship between palbociclib exposure and PFS or the incidence of grade 3-4 AEs was found.

## Introduction

Since 2017, cyclin-dependent kinase 4 and 6 inhibitors (CDK4/6i) have been available for patients with hormone receptor-positive advanced breast cancer. The addition of CDK4/6i (i.e. palbociclib, ribociclib and abemaciclib) to endocrine therapy has nearly doubled progression-free survival (PFS) in both first- and second-line treatment.^1^, ^2^, ^3^, ^4^, ^5^, ^6^ CDK4/6i use is also associated with increased toxicity. Grade 3-4 neutropenia occurs in over 60% of patients treated with palbociclib or ribociclib, and in almost one-quarter of patients treated with abemaciclib.^2^^,^^4^^,^^7^ Other frequently reported adverse events (AEs) include anaemia, thrombocytopaenia, nausea and fatigue.^1^^,^^4^^,^^7^ AEs can lead to treatment interruption, dose modification or discontinuation of the drug, potentially compromising treatment outcomes. Indeed, more than one-third of patients receiving CDK4/6i require a dose reduction due to AEs and almost one-quarter discontinue CDK4/6i treatment early.^8^^,^^9^ Conversely, some patients tolerate the administered dose very well but may not achieve optimal benefit from CDK4/6i therapy as higher doses may be more effective. Given the variability in response and tolerability to CDK4/6i,^1^^,^^2^ patients may benefit from individualised dosing approaches like dose escalations or dose reductions. High interpatient variability in trough plasma levels (*C*_min_) is known.^10^, ^11^, ^12^ However, data on exposure–response or exposure–toxicity relationships of CDK4/6i are limited and inconclusive to date.

In order to reach a greater understanding of the pharmacokinetics (PK) of palbociclib, a population PK model (popPK model) incorporating important predictors for exposure is helpful to predict *C*_min_ and plasma area under the curve (AUC) concentrations from random PK samples. Different popPK models have already been developed to describe the PK and pharmacodynamics of palbociclib.^13^, ^14^, ^15^, ^16^ These models were either based on PK data from clinical trials (phase I, II and III trials) or from real-world data with limited sampling. Interestingly, these different sources of PK data resulted in different popPK model structures to describe palbociclib PK: one-compartment models for real-world data compared with two-compartment models for clinical trial data.^13^, ^14^, ^15^, ^16^ These differences between clinical trial and real-world data may be due to differences in the number of the PK samples available for model development. Based on limited samples, it is often difficult to estimate all PK parameters of a (rather complex) popPK model. An approach taking into account PK information from a previously developed popPK model is beneficial in case of such sparse datasets. This can be achieved using a frequentist Bayesian approach ($PRIOR approach), which enables the estimation of PK parameters based on previous PK data and sparse data collected.^17^ PK data derived from this informed popPK model could help to further delineate the variability in response and tolerability to palbociclib in our cohort.

In this paper, we developed a popPK model of palbociclib using the $PRIOR approach. With this popPK model, we predicted individual *C*_min_- and AUC concentrations from random PK samples taken from patients in the SONIA study. The SONIA study evaluated the efficacy of CDK4/6i added to either first- or second-line endocrine therapy in patients with hormone receptor-positive, human epidermal growth factor receptor 2 (HER2neu) receptor-negative breast cancer. We aimed to investigate the potential relationship between palbociclib exposure and clinical response or toxicity and dose reductions in the SONIA study.

## Materials and methods

All data were derived from patients participating in the SONIA (Selecting the Optimal position of CDK4/6 Inhibitors in HR+ Advanced breast cancer) study. The SONIA study is a multicentre randomised phase III study evaluating the efficacy, safety, quality of life and cost-effectiveness of CDK4/6i added to either first- or second-line endocrine therapy in patients with hormone receptor-positive, Her2neu receptor-negative advanced breast cancer.^18^ The study was approved by the Medical Ethics Committee from the Netherlands Cancer Institute in March 2017 (MEC 17-1120) and carried out according to the Declaration of Helsinki. The study was registered in the European Clinical Trials database (2015-000617-43) and in the ClinicalTrials.gov database (NCT03425838). Patients were included in the study between November 2017 and September 2021 in the Netherlands.

### Study design

Patients were eligible for the study if they were diagnosed with advanced breast cancer and planned to receive aromatase inhibitors as first-line treatment. A total of 1050 patients were randomised 1 : 1 to receive either first-line treatment with an aromatase inhibitor plus a CDK4/6i, followed at progression by fulvestrant monotherapy in second-line or first-line treatment with monotherapy aromatase inhibitor followed at progression by fulvestrant plus a CDK4/6i in second line. A detailed description of the study and the main results has been published earlier.^18^ Patients started with the standard dose of palbociclib 125 mg. Dose reductions were carried out according to the study protocol/summary of product characteristics, i.e. dose reduction was recommended for the subsequent cycles in case a Common Terminology Criteria for Adverse Events (CTCAE) grade ≥3 non-haematological AE occurred. In case of a grade 3 haematological AE, palbociclib dose was only reduced in case of prolonged (>1 week) recovery to grade ≤2, recurrent grade 3 haematological AE or need for transfusions. Palbociclib dose was always reduced in case of a grade 4 haematological AE. In general, a first dose reduction would be to 100 mg/day and a second dose reduction to 75 mg/day. Dose re-escalation was not allowed.

Since February 2018, patients in the SONIA study could consent separately to participate in the PK part of the study. Extra blood samples were taken at random time points on day 15 ± 5 days of cycle 1 and day 15 ± 5 days of cycle 2 during the treatment with a CDK4/6i. Time of blood withdrawal and time of last CDK4/6i intake before blood withdrawal were noted for every patient. Blood was collected in EDTA tubes and processed at the day of collection by centrifugation at 1500-2000 *g* for 10 min. Plasma was stored at −20°C until analyses. Plasma concentrations were quantified using a validated liquid chromatography–tandem mass spectrometry method.^19^

### Population pharmacokinetic model

A popPK model was developed using NONMEM (version 7.5.0, ICON development Solutions, Ellicott City, MD) software. Since only limited sampling data were available for each patient, the $PRIOR subroutine as Bayesian modelling was used to inform poorly informed parameters of our data (based on the distribution of the prior parameter) according to Chan Kwong et al.^17^ This is an elegant alternative to fixing PK parameters in case of sparse datasets due to difficulties estimating all PK parameters. Using the $PRIOR approach enables modelling of sparse data. The previously developed two-compartment model by Courlet et al. was used as a prior information model.^13^ Model development using the prior subroutine is further specified in the Supplementary Data, available at https://doi.org/10.1016/j.esmoop.2025.104290. Finally, a brief covariate analysis was carried out to potentially further improve the model fit, where allometric scaling and differences between capsules and tablets were investigated (see Supplementary Methods, available at https://doi.org/10.1016/j.esmoop.2025.104290 for details).

To discriminate and select between models, physiological plausibility, goodness-of-fit (GOF) plots, precision of parameter estimates and change in objective function were assessed. A significant improvement of the fit for hierarchical nested models was considered at a drop of ≥3.84 points, corresponding to a *P* < 0.05 (chi-square distribution with 1 degree of freedom). The GOF plots and prediction-corrected visual predictive checks (pcVPCs) were assessed to evaluate model fits.^20^^,^^21^

### Pharmacokinetic analyses

For each patient, area under the curve (AUC_0-tau_) plasma concentration and trough levels (*C*_min_) at cycle 1 and 2 were constructed from random samples using the PK model. AUC_0-tau_ describes the palbociclib exposure during the treatment period of 3 weeks, since palbociclib is prescribed in cycles of 3 weeks followed by 1 week off. AUC_0-tau_ was derived from the PK model by dividing the dose by the individual estimated clearance (CL), which was derived using maximum a posteriori Bayesian estimation. Individual PK parameter estimates (i.e. empirical Bayes PK estimates) were also used to obtain individual predicted *C*_min_.^22^ When PK data for both cycles were available, average AUC_0-tau_ and *C*_min_ for both cycles together were calculated per patient. If PK data were available for only one cycle, AUC_0-tau_ and *C*_min_ from this cycle were used for analyses. At least one sample should be above the lower limit of quantification in order for a patient to be included. If a patient switched to another CDK4/6i after the first cycle, only PK data from the initial CDK4/6i (i.e. palbociclib) were used. Since there was only a maximum of two PK samples per patient (spare data sampling), it was not possible to simulate other PK endpoints such as time until maximum concentration (*T*_max_) or peak plasma concentration (*C*_max_) due to the high risk of shrinkage toward the population mean.

### Response analyses

Radiologic response evaluation was carried out every 12 weeks according to RECIST v.1.1.^23^ The relation between exposure and PFS was analysed separately as per treatment strategy. First progression since the start of palbociclib treatment, either in first or second line depending on when the patient received CDK4/6i treatment, was used for exposure–response analysis. Progression was defined as objective disease progression according to RECIST, clinical deterioration on palbociclib leading to discontinuation of therapy, initiation of chemotherapy for breast cancer or death, whichever occurred first.

### Toxicity analyses

Laboratory assessment (haematology and chemistry) was carried out every 2 weeks in the first two cycles, every 4 weeks during cycle 3 and 4 and every 3 months in the cycles thereafter in patients receiving CDK4/6i. Grade 3 and 4 AEs were assessed at every visit according to the CTCAE. To exclude as many competing risks as possible, only grade 3 and 4 AEs in the first 3 months after start of CDK4/6i were included for analyses of the relation between exposure and toxicity. AEs were analysed separately when occurring in at least 10% of patients in the first 3 months of treatment. In patients in whom the dose of palbociclib was reduced in the second cycle due to AEs in the first cycle, only the PK data of the first cycle were included in the exposure–toxicity analyses. Also, when analysing the relationship between exposure and dose reduction, for all patients who underwent dose reduction in the second cycle, only PK data of the first cycle were included.

### Statistical analyses

Baseline characteristics of patients were analysed using descriptive statistics. The primary endpoint for exposure–response analyses was time from randomisation to first progression for patients treated with palbociclib in the first line and time from start of palbociclib until first progression in the second line for patients treated with palbociclib in the second line. Univariable Cox regression was carried out to test the association between PFS and palbociclib exposure by means of *C*_min_ and AUC_0-tau_. For further insight, palbociclib exposure was split above or below median and above or below the first quartile level. Hazard ratios (HRs) were estimated by means of a Cox proportional hazards model. Survival curves were generated by Kaplan–Meier analysis. The occurrence of AEs or dose reductions was compared between different quartiles of palbociclib exposure. A chi-square test for trend in proportions was used to test whether a trend was seen for more AEs or dose reductions with higher palbociclib exposure.

## Results

A total of 652 samples of 366 patients were used for the development of the PK model. For analyses of the exposure–response and exposure–toxicity, only data of patients who initiated palbociclib at least 3 months before the data cut-off (1 December 2022) were included. Therefore, PK data were available for 344 patients, of whom 235 patients were treated with palbociclib in first line and 109 patients, those who progressed to second line before data cut-off, were treated with palbociclib in second line. Baseline characteristics can be found in Table 1.

**Table 1:** Baseline characteristics

Median [IQR] or *n* (%)	Palbociclib in first line (*n* = 235)	Palbociclib in second line (*n* = 109)
Age,^a^ years	63 [54-70]	60 [53-71]
Weight,^b^ kg	73 [65-84]	75 [64-88]
BMI,^b^ kg/m^2^	26 [24-30]	26 [24-30]
WHO performance^a^		
0	106 (45)	55 (50)
1	116 (49)	48 (44)
2	13 (6)	6 (6)
Menopausal status		
Pre- or perimenopausal	37 (16)	19 (17)
Postmenopausal	198 (84)	90 (83)
Visceral disease		
Yes	129 (55)	62 (57)
No	106 (45)	47 (43)
Bone-only disease		
Yes	40 (17)	19 (17)
No	195 (83)	90 (83)
Aromatase inhibitor^c^		
Anastrozole	57 (24)	—
Letrozole	178 (76)	—

BMI, body mass index; CDK4/6i, cyclin-dependent kinase 4/6 inhibitor; IQR, interquartile range; WHO, World Health Organization.

a At the moment of inclusion in the SONIA study.

b At the start of CDK4/6i.

c During CDK4/6i treatment.

### PK model development

Palbociclib PK was adequately captured by a two-compartment model with first-order absorption (including a lag time), where the final popPK model was informed based on a prior model.^13^ From the 652 samples, 51 samples (7.8%) were derived from patients using palbociclib tablets instead of capsules. No difference in bioavailability between capsules and tablets was identified. However, allometric scaling improved the GOF plots and was added to all relevant model parameters [CL, central compartment (V1), peripheral compartment (V2) and intercompartmental CL between V1 and V2 (Q)] with fixed allometric scaling components (0.75 for CL and Q and 1 for V1 and V2). CL was estimated at 62.6 l/h [relative standard error (RSE) 2%] and a high volume of distribution was estimated for both compartments [2370 l (RSE 7%) and 682 l (RSE 8.5%) for V1 and V2, respectively]. All PK parameters were estimated with good precision (RSEs < 31%). Final parameter estimates and further information regarding model development are provided in the Supplementary Results, available at https://doi.org/10.1016/j.esmoop.2025.104290. In addition, the GOF plots and pcVPCs of the final model are shown in Supplementary Figures S1 and S2, available at https://doi.org/10.1016/j.esmoop.2025.104290.

### Exposure–response analyses

**Figure 1:**
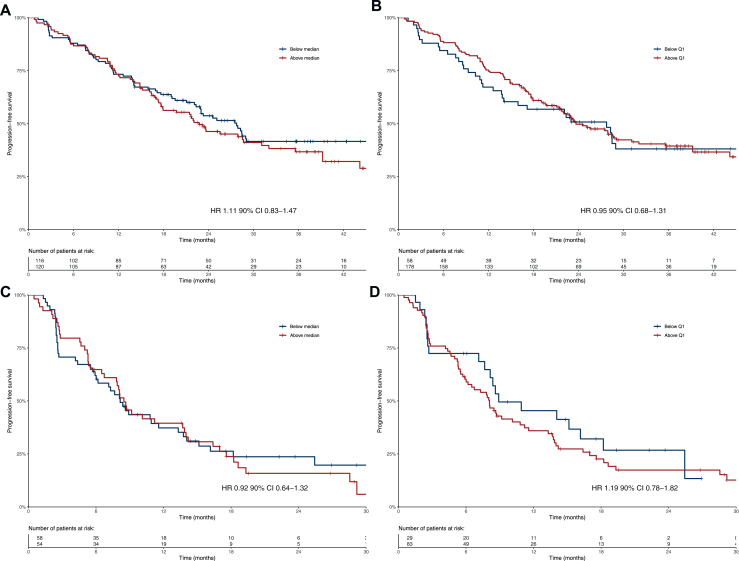
**Kaplan–Meier curves of exposure–response relationship of palbociclib.** Palbociclib trough levels were included in the analyses. (A, B) Progression-free survival when palbociclib was given as first-line treatment. (C, D) Progression-free survival when palbociclib was given as second-line treatment. CI, confidence interval; HR, hazard ratio; Q1, first quartile.

For 270 patients, PK data of both treatment cycles were available, for 55 patients only PK data of cycle 1 were available and for 19 patients only PK data of cycle 2 were available. Thirty patients used a lower dose of palbociclib in cycle 2 (100 mg, *n* = 26; 75 mg, *n* = 4). The median *C*_min_ of palbociclib during cycle 1 and 2 in our cohort was 60 ng/ml [interquartile range (IQR) 50-74 ng/ml, min-max 16.1-130.8 ng/ml] and the median AUC_0-tau_ was 1886 ng∗h/ml (IQR 1650-2210 ng∗h/ml, min-max 1007-3567 ng∗h/ml). In first line, there was no significant association between PFS and palbociclib exposure [HR 0.98 per 10 units (ng/ml) increase, 90% confidence interval (CI) 0.90-1.06, *P* = 0.60 for *C*_min_ and HR 1.00, 90% CI 0.96-1.03 per 100 units (ng∗h/ml) increase, *P* = 0.91 for AUC_0-tau_]. Also, PFS did not differ significantly between patients with palbociclib *C*_min_ above or below median (HR 1.11, 90% CI 0.83-1.47) or between patients with palbociclib *C*_min_ above or below the first quartile limit, i.e. 50 ng/ml (HR 0.95, 90% CI 0.68-1.31). For the latter analysis, the Kaplan–Meier curves crossed but seemed to show an effect early on. This was checked by means of the restricted mean survival time method, yet again no effect could be found. Similar results were found for patients treated with palbociclib in second line (Supplementary Table S1, available at https://doi.org/10.1016/j.esmoop.2025.104290). Kaplan–Meier curves and HRs can be found in Figure 1. When the analyses were repeated using AUC_0-tau_, similar results were found (Supplementary Table S1, available at https://doi.org/10.1016/j.esmoop.2025.104290).

### Exposure–toxicity analyses

Twenty-four of 344 patients experienced toxicity grade 3-4 during cycle 1 leading to a dose reduction in cycle 2. For these patients, only PK data of cycle 1 were included. Since for two patients no PK data at cycle 1 were available, toxicity analyses were carried out using 342 patients. One hundred and ninety-one of 342 (56%) patients experienced any toxicity grade 3-4 during the first three cycles. Neutropenia was the only individual AE occurring in >10% of patients during the first three cycles of treatment, occurring in 155 of 342 (45%) patients. Patients were divided in quartiles according to *C*_min_ and AUC_0-tau_ concentrations. Because of exclusion of some PK data for the toxicity endpoint, quartiles of palbociclib PK were slightly different compared with the exposure–response analyses. The occurrence of AEs was compared between different quartiles and can be found in Table 2. No significant trend for more AEs with higher palbociclib exposure could be found. No other individual toxicities could be analysed separately because of too low incidence in the first 3 months of treatment.

**Table 2:** Adverse events and dose reduction in different palbociclib exposure groups

Palbociclib *C*_min_	Q1 < 50.8 ng/ml	Q2 50.8-60.8 ng/ml	Q3 60.9-74.7 ng/ml	Q4 ≥ 74.8 ng/ml	*P* for trend
All AEs grade ≥3	45/86 (52%)	52/85 (61%)	46/85 (54%)	48/85 (56%)	0.88
Neutropenia grade ≥3	35/86 (41%)	39/85 (46%)	40/85 (47%)	41/86 (48%)	0.36
Dose reduction	25/83 (30%)	39/87 (45%)	36/85 (42%)	44/86 (51%)	0.01^a^

**Palbociclib** **AUC**_**0-tau**_	**Q1 < 1660 ng∗h/ml**	**Q2 1660-1900 ng∗h/ml**	**Q3 1900-2220 ng∗h/ml**	**Q4 ≥ 2220 ng∗h/ml**	***P* for trend**

All AEs grade ≥3	43/86 (50%)	50/85 (59%)	52/85 (61%)	46/86 (53%)	0.59
Neutropenia grade ≥3	31/86 (36%)	38/85 (45%)	46/85 (54%)	40/86 (47%)	0.09
Dose reduction	20/83 (24%)	41/87 (47%)	40/85 (47%)	43/86 (50%)	0.001^a^

AE, adverse event; AUC, area under the curve plasma concentration; *C*_min_, trough concentration; Q, quartile.

a Statistically significant.

Additionally, the incidence of dose reductions during the entire treatment period with palbociclib was compared across different quartiles of exposure. Here, for all patients who had already received a dose reduction in cycle 2, only PK data from cycle 1 were used. This applied to 30 patients, 3 of whom had no PK data available for cycle 1, resulting in 341 patients available for analysis. All but three dose reductions were due to some form of toxicity (not further specified). There was a significant relationship between higher palbociclib *C*_min_ (*P* = 0.01) and AUC_0-tau_ (*P* = 0.001) and the occurrence of dose reductions (Table 2).

## Discussion

In the largest clinical PK study conducted thus far, we found no relationship between palbociclib exposure and PFS. Interestingly, no relationship between palbociclib exposure and grade 3-4 AEs in the first 3 months was found. However, patients with higher palbociclib exposure more frequently underwent dose reduction.

For the development of the palbociclib popPK model used in our study, the $PRIOR subroutine was used (adding PK information of a previously developed popPK model) as an alternative to fixing parameters or fitting an unrealistic simplified model due to limited sampling. Different studies have shown that using a prior approach results in a better fit than fixing parameters.^24^^,^^25^ However, a disadvantage of the prior approach is that a covariate analysis cannot be carried out on prior informed parameters.^17^ Since it is known that the time until maximum plasma concentration of palbociclib capsules is longer than that of palbociclib tablets,^26^ we tested the effect of formulation on the relative bioavailability (with no prior weight) to further improve the model fit. However, no effect of differences between capsules and tablets on this relative bioavailability was identified, indicating that formulation does not influence the bioavailability in patients.

Earlier research regarding the relationship between palbociclib exposure and PFS remained inconclusive. For instance, in the PALOMA-1 study, a trend for longer PFS in patients with palbociclib concentration >60 ng/ml, the same cut-off point as in our study, was found but this was not statistically significant and this study was carried out in a small subgroup of patients (*n* = 81, median PFS of 24.5 months versus 17 months).^27^ Another study used popPK modelling to elucidate the relationship between palbociclib plasma concentration and PFS and found no difference between PFS in patients with palbociclib plasma concentrations above or below 78 ng/ml, coinciding with the third quartile limit in our study, in the PALOMA-3 study.^28^ *In vitro*, the concentrations of palbociclib required to achieve 50% inhibition (IC_50_) of CDK4 and CDK6 were found to be 33.5 ng/ml and 48.7 ng/ml, respectively.^11^^,^^29^ Given that these levels are comparable to the average plasma *C*_min_ concentrations observed clinically, we hypothesised that palbociclib efficacy could be influenced by variations in exposure. However, the lack of association between palbociclib exposure and PFS in our and other studies demonstrates that *in vitro* findings do not always directly translate to clinical outcomes.

In contrast with our findings, earlier research regarding the relationship between palbociclib exposure and toxicity did suggest more AEs with higher palbociclib levels. Phase I studies and PK modelling suggested that higher palbociclib concentrations were associated with a higher incidence of neutropenia grade 3-4.^15^ Also, a small prospective study (*n* = 58) found a trend for higher palbociclib *C*_min_ in patients with neutropenia grade 3-4 compared with patients without neutropenia grade 3-4 in the first two cycles (76.7 versus 66.7 ng/ml, *P* = 0.06).^30^ Compared with our study, these studies were small, which might be the reason for the conflicting results.

We showed that patients with higher palbociclib exposure more frequently underwent dose reductions. Almost all dose reductions were caused by some form of toxicity. However, there was no higher incidence of grade 3-4 AEs in the first 3 months of treatment in patients with higher palbociclib exposure. There are several potential explanations for this apparent contradiction. For instance, a substantial burden of grade 1-2 AEs might have prompted the need for dose reduction over time. Unfortunately, these lower-grade AEs were not systematically monitored during the study. Furthermore, our toxicity analyses did not include grade 3-4 AEs occurring beyond the initial 3 months of therapy. Although it is known that most AEs occur in the first 3 months,^31^ it remains unknown if grade 3-4 AEs occurring after this timeframe have influenced the likelihood of dose reduction.

Our results regarding a relationship between palbociclib exposure and dose reduction and the absence of a relationship between palbociclib exposure and PFS suggest that it is safe to reduce the dose in patients suffering from palbociclib-related toxicity. Indeed, a real-world study involving 70 patients, of whom 40 underwent a dose reduction of palbociclib, found no differences in PFS between patients who underwent a dose reduction compared with those who did not.^32^ Similar results were found in real-world studies of ribociclib and abemaciclib.^9^^,^^33^^,^^34^ However, this study does not address whether dose reductions could be safely applied to all patients, including those without toxicity or even from start of treatment, which warrants further investigation. Additionally, it would be interesting to explore a potential relationship between palbociclib exposure and lower-grade or long-term AEs.

Unfortunately, we only had PK data of cycle 1 and cycle 2 in this study. Therefore, dose reductions, interruptions or early discontinuation of treatment occurring after cycle 2 could not be taken into account when comparing PFS across different palbociclib exposure groups. This limitation may have introduced some bias, but it aligns with clinical practice where therapeutic drug monitoring would be most beneficial around start of treatment to consider early dose adjustments. Another limitation is that we had to predict *C*_min_ and AUC_0-tau_ concentrations instead of taking blood samples at the time of minimum concentration or conducting more frequent sampling for each patient.

In conclusion, our study did not find a relationship between palbociclib exposure and PFS. Patients with higher palbociclib exposure more frequently underwent dose reductions. Yet, this was not reflected by a higher incidence of grade 3-4 AEs in the first 3 months. Although more research regarding lower-grade AEs and the effect of dose reductions on efficacy is needed, our results suggest that the dose can safely be reduced in patients who experience palbociclib-related toxicity.
