# Supplementary material for: Palbociclib exposure in relation to efficacy and toxicity in patients with advanced breast cancer
Source: ESMO Open. 2025 Feb 15;10(3):104290. doi: 10.1016/j.esmoop.2025.104290 (PMC11872518; doi:10.1016/j.esmoop.2025.104290)
Supplement: Supplementary Data [file mmc1.docx]

**SUPPLEMENTARY MATERIAL**

**DATA METHODS**

**Model development**

A $PRIOR subroutine (using NWPRI) was used for model development [1]. First, a non-informative model was tested by adding the reference values of the reference model on THETA priors, OMEGA priors, and SIGMA priors [2]. The variance of THETA priors was fixed to 1000, for the degrees of freedom of OMEGA prior was fixed to 4, and for the degrees of freedom of SIGMA prior was fixed to 2. Secondly, an informative prior approach was tested for model development. For this, the variance matrix from the reference model was used for the variance of THETA priors, and the degrees of freedom of OMEGA^2^ prior and SIGMA^2^ prior were calculated according to Chan Kwong *et al.* [1].

The reference model used ADVAN4 and TRANS4 subroutine for model fitting. The final model was a two-compartment model with first-order absorption including a lag time, the absorption constant (Ka) was estimated at 0.8 hour^-1^ and lag time was estimated at 2.0 hours [1].

**Covariate analysis**

Stepwise covariate modelling on parameters estimated with priors could not be performed. Still, two aspects were tested to further improve the model fit. First, the effect of different dosage forms (capsules vs tablets) on the relative bioavailability (F1) was investigated:

*F_1_ =* ${P_{pop}*}^{dosage form}$ (1)

where *F_1_* is the relative bioavailability, *P_pop_* is the typical population parameter estimate and dosage form was 0 for capsules and 1 for tablets.

Secondly, allometric body weight scaling was added with fixed exponents of 0.75 for clearance parameters and 1 for volume of distributions.

**REFERENCE**

1. Chan Kwong AHP, Calvier EAM, Fabre D, Gattacceca F, Khier S. Prior information for population pharmacokinetic and pharmacokinetic/pharmacodynamic analysis: overview and guidance with a focus on the NONMEM PRIOR subroutine. J Pharmacokinet Pharmacodyn. 2020;47(5):431-46. doi: 10.1007/s10928-020-09695-z.

**SUPPLEMENTARY MATERIAL**

**DATA RESULTS**

**Model development and evaluation**

Adding prior as non-informative or informative did not significantly different (difference in objective function values (dOFV): 0.553), also in the Goodness Of Fit (GOF)-plots no difference were seen. Therefore, the informative prior model was used.

Adding allometric scaling and differences in dosage form did not improve the model (dOFV 2.3 and 0.098). However, adding allometric scaling improved the GOF-plots and thus was added to the final model. Variability on Ka was relatively high, probably due to limited samples in the absorption phase. Therefore, in the final model the inter-individual variability on Ka was fixed to the prior model estimate. The parameter estimates of the final model are shown in **Supplemental** **Table 1**. The final model adequately described observed palbociclib data, which is shown in **Supplemental** **Figure 1 and 2**.

**Figure 1. Goodness-of-fit plots of the final model.**


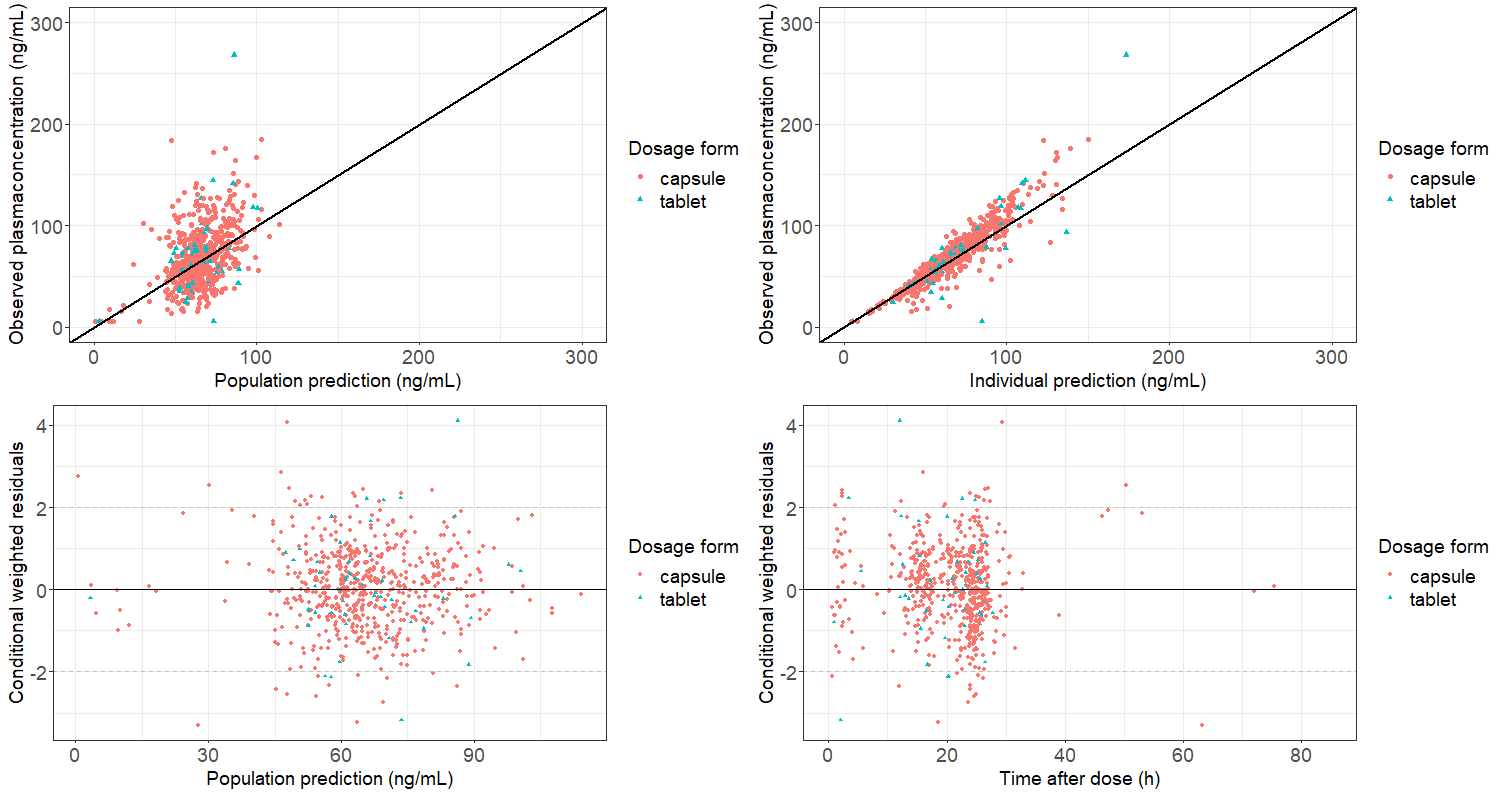
*Observed versus population-predicted palbociclib concentration; observed versus individually predicted palbociclib concentration; conditional weighted residuals (CWRES) versus population-predicted concentrations; and CWRES versus time after dose*

**Figure 2. Prediction-corrected visual predictive check of the final model (*n*=1000).**


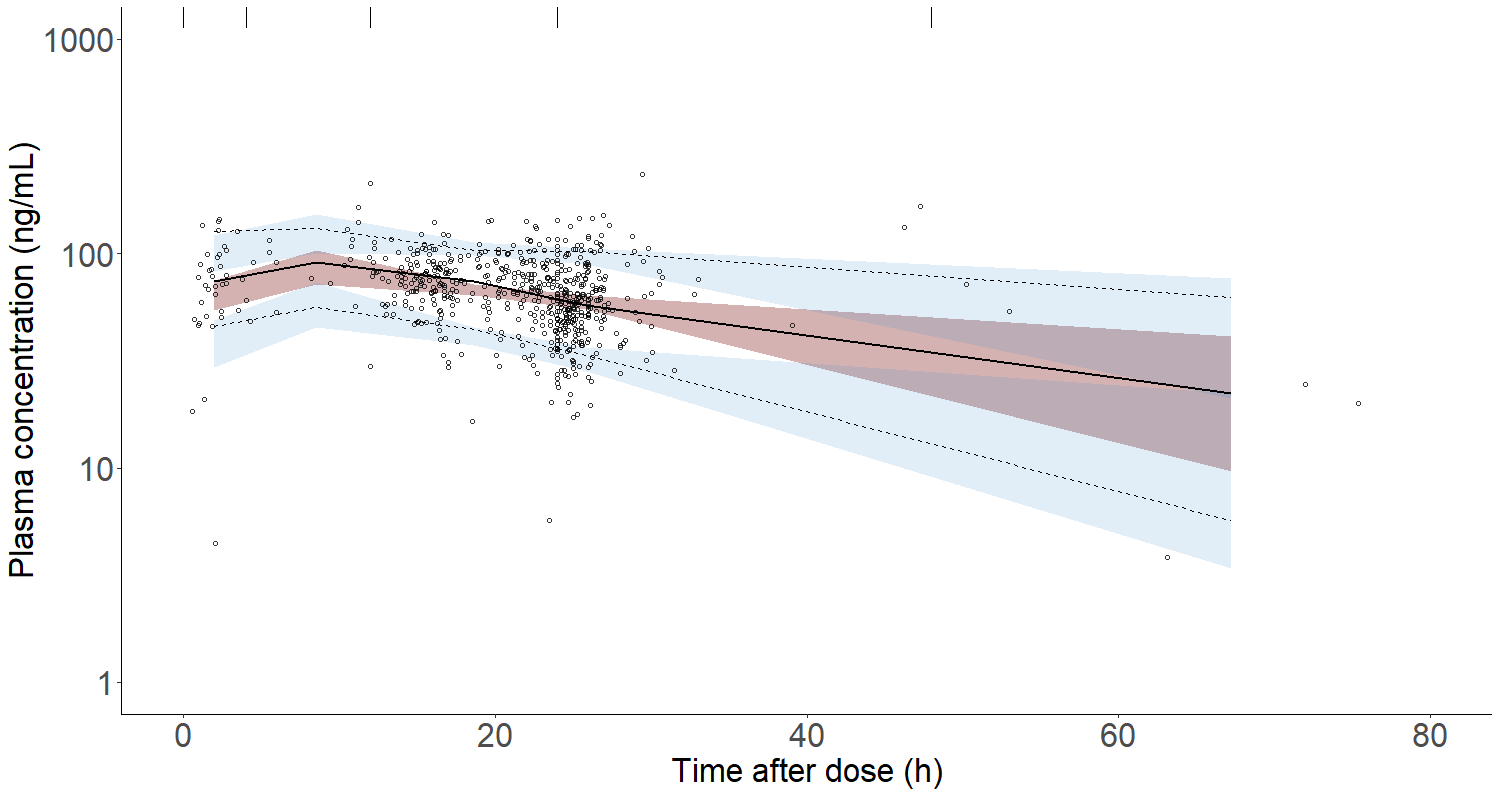


*The solid black line represents the 50th percentile of observed concentrations and the dashed black lines represent the 10th and 90th percentile of observed concentrations. The blue and red areas represent the 95% confidence intervals of the simulated percentiles.*

**Table 1: Final population PK parameter estimates of the prior model generated with inclusion of allometric scaling^#^**

| Parameter (unit) | *Estimate* | *RSE (%)* |
| --- | --- | --- |
| CL (L/h) | 62.6 | 2 |
| V1(L) | 2370 | 7 |
| Q (L/h) | 4.99 | 30.9 |
| V2 (L) | 682 | 8.5 |
| Ka (h^-1^) | 0.941 | 22.6 |
| ALAG (h) | 1.89 | 6.3 |
| IIV CL (CV%) | 24.6 | 6.1 |
| IIV V2 (CV%) | 55.8 | 14.1 |
| IIV Ka (CV%) | 125.2 (fixed) | - |
| Proportional residual error (%) | 20.9 | 4.4 |

*CI = confidence interval; RSE = relative standard error; CL = clearance; V1 = volume of distribution of the central compartment; Q = intercompartment clearance between V1 and V2; V2 = volume of distribution of the peripheral compartment 2; IIV = interindividual variability. ^#^The estimation in this table is of a typical patient of 70 kg.*

**Code of the final model**

$SUBROUTINES ADVAN4 TRANS4

$PRIOR NWPRI

$PK

; PK parameters

TVCL=THETA(1)*((WT/70)**THETA(7))

CL = TVCL*EXP(ETA(1))

TVV2=THETA(2)*((WT/70)**THETA(8))

V2 = TVV2*EXP(ETA(2))

TVKA = THETA(3)

KA= TVKA*EXP(ETA(3))

Q=THETA(4)*((WT/70)**THETA(7))

V3=THETA(5)*((WT/70)**THETA(8))

ALAG1= THETA(6)

S2 = V2/1000

$ERROR

IPRED = F

Y = F + F * ERR(1)

$THETA

63 ; CL

2480 ; V2

1.01 ; KA

6.13 ; Q

690 ; V3

1.89 ; ALAG

0.75 FIXED ; WT CL and Q

1 FIXED ; WT V

$OMEGA BLOCK(2)

0.0653 ; ETA CL

0 0.0146 ; ETA V2

$OMEGA

0.943 FIXED ; ETA KA

$SIGMA

0.0398 ; prop error PK

$THETAP

67 FIXED ; CL

2800 FIXED ; V2

0.8 FIXED ; KA

7 FIXED ; Q

704 FIXED ; V3

2 FIXED ; ALAG

$THETAPV BLOCK(6) ; informative

1.01E+01 FIXED

1.00E+02 4.20E+04

-3.11E-01 1.02E+00 6.50E-02

4.36E-01 9.28E+01 7.18E-03 3.98E+00

6.48E+00 9.64E+02 -1.48E+00 7.86E+01 4.05E+03

-6.66E-02 1.08E+00 1.64E-02 3.29E-02 2.91E-01 2.36E-02

$OMEGAP BLOCK(2) ; informative

8.31E-02 FIXED

0.00E+00 9.94E-02

$OMEGAPD ; informative

7.35 FIXED

$SIGMAP

0.03 FIXED ; prop error PK

$SIGMAPD

2 FIXED ; non-informative

$EST METHOD=1 INTER MAXEVAL=2000 NOABORT PRINT=1

$COV MATRIX = R PRINT=E

**Supplementary Table 1**

|  | **Palbociclib in first line** | **Palbociclib in second line** |
| --- | --- | --- |
| **C_min_** | | |
| **Continuous (per 10 units)** | HR 0.98, 90% CI 0.90-1.06 | HR 0.95, 90% CI 0.88-1.03 |
| **Median split** | HR 1.11, 90% CI 0.83-1.47 | HR 0.92, 90% CI 0.64-1.32 |
| **Q1 split** | HR 0.95, 90% CI 0.68-1.31 | HR 1.19, 90% CI 0.78-1.82 |
| **AUC** | | |
| **Continuous (per 100 units)** | HR 1.00, 90% CI 0.96-1.03 | HR 0.98, 90% CI 0.95-1.02 |
| **Median split** | HR 1.19, 90% CI 0.90-1.58 | HR 1.01, 90% CI 0.71-1.44 |
| **Q1 split** | HR 1.04, 90% CI 0.75-1.46 | HR 1.07, 90% CI 0.70-1.61 |

*C_min_: trough level, AUC: area under the curve plasma concentration, HR: hazard ratio, CI: confidence interval*
